# Achieving Sustainable Development Goals (SDGs) in Sub-Saharan Africa (SSA): A Conceptual Review of Normative Economics Frameworks

**DOI:** 10.3389/fpubh.2020.584547

**Published:** 2020-11-16

**Authors:** Michael E. Otim, Amina M. Almarzouqi, Jean P. Mukasa, Wilson Gachiri

**Affiliations:** ^1^University of Sharjah, College of Health Sciences, Sharjah, United Arab Emirates; ^2^Nexus International University, College of Graduate and Research, Kampala, Uganda; ^3^Fatima College of Health Sciences, Ajman, United Arab Emirates; ^4^The Birmingham Leadership Development Centre, Dubai, United Arab Emirates

**Keywords:** normative frameworks, social justice, equity, priority setting, resource allocation, Sub-Saharan Africa (SSA), sustainable development goals (SDGs)

## Abstract

**Background:** The health status of the Sub-Saharan African (SSA) countries is well below that of the rest of the world. Coupled with low per capita income, these countries have agreed and committed themselves to raising their health status equitable standard by addressing United Nations (UN) Sustainability Development Goal number 3 (SDG3) by 2030. Addressing SDG3 requires increased and equitable funding for universal health coverage, healthcare infrastructure, efficient resource allocation, improved priority setting, reduction in corruption, and other strategies. However, what is urgently needed to improve priority setting processes or meaningful health system reform, among other things. There is therefore a need for the exploration of the economic and non-economic (which includes social justice) explicit criteria that ought to form the normative framework for Decision Making. These explicit criteria include efficiency, burden of disease, equality (strict egalitarianism), equity, and explicit criteria.

**Methods:** The ultimate aim was to identify explicit values/principles/criteria that can be used to formulate an ideal normative framework to be used to guide decision Making so as to improve SDG3 in SSA. We synthesized selected literature on the normative frameworks for priority setting processes in health in SSA was undertaken, and the explicit criteria which, ought to guide these frameworks were identified. The form of the Social Welfare function and its principles was identified.

**Results and Conclusions:** The framework and its explicit criteria for priority setting in the SSA countries that ought to be adopted in order to improve their SDG3 was identified—Non-Welfarist framework. This framework allows utility, health and other important social values/attributes/principles to enter the normative SWF. It is argued that such a framework ought to be specified empirically and concurrently by the decision-makers and members of the community representatives. Community representatives ought to be recognized as legitimate claimants of the resources determined, and should therefore be allowed to have a role in specifying the arguments in the SWF and what weights to be attached to the stated arguments. This implies that the selection of options in decision-making should focus on maximizing benefit and minimizing the opportunities forgone as stated in the framework.

## Background

Sub-Saharan African (SSA) countries have significantly poorer health outcomes than the rest of the world in spite their efforts at addressing Millennium Development Goals (MDGs). Later, the United Nations (UN) developed the Sustainable Development Goals (SDGs) to replace the MDGs, with Goal number 3 focusing on health (SDG3). SSA countries signed up and pledged to achieve the specified targets by 2030 while addressing the needs of all persons with disabilities (1). All countries that have signed up to the SDGs aim to achieve goals such as neonatal mortality rates to 12 per 1,000 live births and under-5 mortality rate to at least 25 per 1,000 live births, Non-Communicable Disease (NCDs) and other SDG3 sub-goals. These countries have pledged to increase their investments in health to improve health outcomes such as the reduction of the global maternal mortality indicator (to <70 per 100,000 live births); and end preventable deaths of newborns and children under 5 years of age (1). However, SSA countries face several inequities within themselves and in comparison, with other countries.

SSA is a diverse group by population, size, and income levels. They are economies, according to World Bank, that include countries with gross national income per capita between $1,006 and $3,955 per capita and those between $3,956 and $12,235 (2). The World Bank further reports that SSA account for the 75% of the world's population and 62% of the world's poor. Thirty percentage of the global domestic product and are potentially significant engines of global growth.

The strategies to achieve SDG3 in SSA countries may include the following (1):

1. Addressing the funding issues for providing health care services and enabling people's access to health insurance for services.
2. Employing other strategies that SSA countries ought to employ such as addressing structural challenges in the health care systems and strengthening the human resources for health.
3. Strengthening country's capacities for early warning and awareness, risk reduction and management of national and global health risks.
4. Lastly but not least, SSA countries improving the Decision Making (priority setting) frameworks so as to invest the scarce resources efficiently and equitably, and what normative framework ought to be used to appropriately to inform such decision making.

This paper focuses on the last point above—improving the decision making process from an economics perspective, whilst recognizing the role of non-economic frameworks given that, markets alone fail to allocate resources optimally and in socially desirable manners. From an economics perspective, it is important to maximize health outcomes (SDG3) for the given investments, whereas losers from such investments ought to be compensated, and the community ought to be involved in the decision making process to enhance transparency and accountability. Two major options exist: either the healthcare systems should be reformed and/or; the decision-making (priority-setting) process by government should be improved. Improvement in priority setting ought to include: the use of evidence; equitable allocations of scarce resources; and transparent processes through an appropriate normative framework.

This study aims to develop the explicit criteria or principles to guide decision-making, and develop normative frameworks for Priority Setting as a means to achieve SDG3. Decision making or Priority setting is defined here as a non-market based economic mechanism of allocating resources or “identifying who gets what at whose expense” (3). These strategies are essential for creating physical, social, and policy environments that will sustain and enhance health and well-being. The combination of targets under SDG3 require inter-sectoral collaborations with interdisciplinary approaches. Of course, it is imperative that these countries reform their health systems to improve services delivery and increase financing (such as universal health coverage) to target SDG3—an issue to be investigated later.

The justification for focusing on priority setting in SSA can be traced, to the evidence of failure for markets alone to allocate resources optimally to improve outcomes to achieve SDG3. First, there is clear evidence of inefficiencies in sectors of health systems. Second, expenditure in health does not seem to reflect the scale of the health problem, since access and health coverage is not adequate—hence the call for universal coverage. Finally, priority-setting exercise is often implicit and therefore lack transparency in the allocations and trade-offs of those allocations. Thus, the need for an explicit process. The key challenge however is how to explicitly specify a normative framework, which ought to guide explicit priority setting in health in the SSA given its context. The research question was: What does the explicit normative framework to guide priority setting in SSA look like and what explicit criteria ought to inform it?

Objectives to be addressed included:

- To identify the economic principles which underpin priority setting?
- To identify the forms of the Social Welfare Functions (SWF), which are suitable in the development of the framework for Priority Setting. Social welfare refers to the overall economic welfare of society, and it can be specified using specific principles, with strong assumptions, as the summation of the welfare of all the individuals in the society (4).
- Identify the principles or values that ought to enter the SWF for priority setting.
- Specify the SWF which embodies or has potential to embody non-economic principles such as equity and social justice which ought to guide priority setting.
- Finally, we then present the normative framework which ought to guide SSA in their efforts to achieve SDGs.

## Methods

This paper draws knowledge from the theoretical frameworks in economics, health economics, and social justice. The ultimate aim was to identify the explicit criteria that can be used to formulate an ideal normative framework to be used to guide priority-setting so as to achieve SDG3 in SSA.

We searched the electronic databases such as Medline, CINAHL, PubMed, EMBASE, PubMed Central (PMC), and Library book collections for relevant books, book chapters, and peer-reviewed articles. We also did a Google search of the internet in general. The following key words: resource-allocation, normative economics, priority-setting, and values/principles, were used to search the databases and the Internet. Studies done in Low and Middle Income Countries (LMIC), social justice and health, and others were selected and studies done in high income countries were excluded.

The top 200 results from the databases and Google searches were further screened for the relevance. The synthesis of the literature on the normative frameworks for priority setting processes in health in SSA was undertaken, and the principles which, ought to guide these frameworks were identified. The form of the Social Welfare function and its principles was then developed.

## Contribution From Economic Foundations for P/S

To achieve SDG3 in SSA, it is critical to identify explicit principles that ought to guide the decision making process. Such principles could involve effectiveness, equity, equality, opportunity costs, and efficiency of interventions and health outcomes to be built into the normative economic framework to drive the priority setting process. It is important therefore, to develop a conceptual normative framework that makes use of such explicit principles to achieve SDG3 in SSA from an economic and social justice perspectives.

### Resource Allocation From an Economics Perspective

The foundation of economics is opportunity cost—the benefits of alternative policy actions taken due to choices made in allocations of limited resources. Opportunity cost principles are recognized, on the basis that society's need/wants are infinite yet the resources to address those needs are limited or scarce, constituting the second key principle of economics for PS. It is the notion that, resources for addressing needs and insatiable wants are scarce, and so decision-makers have to make choices or trade-offs. Trade-offs inevitably generate losers and winners from an economic activity. The losers have to be compensated in principle. Decision-making from an economic perspective occurs at the margin as incremental change (hence marginal analysis), and ought to minimize losses and/or maximize gains from the resources allocated (efficiency) (5, 6). Marginal analysis (MA) is a tool for generating efficiency.

Normative economics is often referred to as welfare economics, and uses microeconomic techniques to simultaneously determine allocative efficiency and the income distribution associated with it (4, 7). It links the competitive market mechanism with Pareto optimality, by stipulating a social welfare improvement from one inferior social state to a superior one. A competitive market allows for resource allocation maximization or Pareto optimality to be achieved, allowing an invisible hand of competition to transform private greed into social welfare (5, 8–10). A competitive market assumes that there are infinite number of self-interested agents, they freely enter and exit the market, and have full information about the goods and services. It also assumes that agents pay a full price for their consumption of the normal goods and services in the market (4, 11). When a competitive market exists, the competitive equilibrium occurs via marginal analysis, ensuring that maximization or Pareto optimality is achieved (5, 9, 12, 13). Thus, a competitive market equilibrium ensures that maximization or Pareto optimality is achieved, allowing an invisible hand of competition to transform private greed into social welfare. However, when the above conditions for perfect competition are not present, markets alone fail to allocate resources optimally and equitably in health care (Market Failure).

### Justification for Government Involvement in Resource Allocation in Health Care

When market failure occurs in the health, government intervention becomes necessary. SDG3 in particular, is prone to market failure due to information asymmetry and lack of access to healthcare (10, 14–17). Further, market failure occurs due to: people's inability to pay for the services; information asymmetry between consumers and providers; externalities; monopoly power; and pure public goods in the market (6, 10, 14–16).

People's inability to pay for healthcare determines one's access to such services, thus justifying government involvement in the healthcare market. For example, the UN is helping Sub-Saharan African Countries to access funding or loans from different sources to ensure that their citizens have universal health coverage (1). Furthermore, information asymmetry between patients and healthcare providers is inherent in health, since doctors having better information about the consumers' health, health care interventions and risk factors than the patient (8). This asymmetry violates the consumer sovereignty. So, privately determined consumption may lead to inefficient allocations, providing justification for government intervention.

Another important issue is that of externalities. This is when one consumer's actions affects the well-being of other consumers, resulting in market failure. In order to correct these externalities, government intervenes either to restore market competition or takes over the resource allocation (6, 13, 14). Another cause of market failure in health is the concept of pure public goods, a class of goods which private providers may not be able to supply adequately. These goods are characterized by non-excludability and non-rivalry in their consumption (6, 15). In these cases, improving SDG3 in SSA becomes a responsibility of the government.

Equity is another case where the market allocation cannot address due to the uniqueness of health as a good, hence market failure. Equity involves the ethical judgments about the fairness of the distribution of the costs and benefits of the health outcomes in the community, the fairness of the process of allocation (due process), or identification of the gainers and losers (4, 6, 17–19). To achieve equity in health in SSA requires government intervention, and this may require evidence for policy making to improve decision making, reform of the health systems, and universal health coverage. The challenge however, is how such evidence can be specified and incorporated in the *t* decision-making frameworks and models—a task addressed in this paper. However, challenges exist on how to use economic principles in decision making.

### Social Welfare Functions

Given the challenges of choosing between alternative options from an allocation, the achievement of the social optimum shifted to the SWF. The SWF is a function which ranks social states as less desirable, more desirable, or indifferent for every possible pair of social states to aid decision making. Several SWFs have been proposed under the N-C welfarist framework and may include the Bergson-Samuelson SWF, and the axiomatic SWF of Ng, Fleming, Harsanyi, and Nash (4, 7, 11, 20). One popular N-C welfarist SWF is the utilitarian social welfare function, also called a Benthamite welfare function (17). This SWF sums up the utility of each individual in order to obtain society's overall welfare. All people are treated the same, regardless of their initial level of utility. One extra unit of utility for a starving person is not seen to be of any greater value than an extra unit of utility for a millionaire. The N-C Welfarist SWF can be defined as a function of only individual utilities/welfares (4, 7, 11, 20):

$$\begin{array}{l} \text{SWF}=\text{f}\left({\text{U}}_{1},{\text{U}}_{2}\ldots {\text{U}}_{\text{i}}\right)\text{ or SWF}\\ \;=\text{f}\left({\text{U}}_{1}\right)+\text{f}\left({\text{U}}_{2}\right)+\ldots \ldots +\text{f}\left({\text{U}}_{\text{i}}\right) \end{array}$$

Where U_i_ stands for the utility/satisfaction/happiness of the ith individual.

Thus, individual consumer preferences/utilities would be elicited from the individuals and then aggregated to form one social preference or health constitution (21). Under normative economic frameworks, maximizing the social preference or outcomes for the given resource allocation (efficiency). Thus, the key criterion to be incorporated in an economic normative framework. Under the perfect market mechanism resource allocation occurs at desirable level—equitable distributions automatically occur. However, when market failure occurs, the free market allocation fails to achieve equity as a criterion.

### Non-economic Principles

Equity and other social justice principles are unfortunately not clearly articulated in economics, and therefore tend to be left out in normative economic frameworks. Equality for example, is the key principle in egalitarianism and in the Rawlsian theories of social justice. Rawls stated that it is “a standard whereby the distributive aspects of the basic structure of society are to be assessed” (22). According to Rawls, the principles of justice are manifested as part of the social contract that is chosen by free and rational human beings who are behind the “veil of ignorance” of their own places in society. Under Rawls's theory, the equality principle may be expressed as equal rights, liberties, health, and/or opportunities.

The equality principle signifies a relationship between groups of persons that have similar qualities in at least one respect, such as equality of health outcomes. It may be expressed as “equality in outcomes,” “equity,” “equality in opportunities,” “equality in access or use,” “equal human rights,” or “equal treatment for equal need” (23). Equity principle is a generally used principle in economics, however, it is not clearly articulated. The equality principle contrasts with the equity principle, in that the equality principle seeks to satisfy all needs regardless of effort and socio-economic status. Equity principle may emphasize distributive justice at the expense of procedural justice issues.

In whatever way it is defined, equity principle has been a dominant reason for government intervention in the health sector to reduce inequalities in population health, to achieve a fair and just process of allocation (24). It is generally defined in economics as equality in the distribution of some phenomena in a socially desirable way such as wealth, rights, etc. but with some added qualification such as “equality of need” (25–28). Two approaches are being used by several countries and SSA countries are attempting translate equity principles into practice (29): horizontal equity and vertical equity. Horizontal equity assumes that the groups or individuals being addressed have equal economic and social standards, and therefore their health needs or the ability to pay is the same. The application of horizontal equity is more suited, to a limited extent, to the allocation of resources within countries or regions in the SSA communities themselves. Health problems between SSA communities themselves are highly disparate, such as between rural and remote communities areas (29). It would not seem appropriate to assume that these two groups have the same health needs. The use of horizontal equity criterion between communities vis-à-vis the rest of the country, would be inequitable and unfair. Vertical equity would be the appropriate principle. Vertical equity refers to the notion of unequal but equitable treatment. The vertical and horizontal equity principles would enter the social welfare function.

The equity principle explains the role of justice in social interactions and priority setting that may be motivated by both self-interest and the desire to address perceived inequities. According to the advocates of equity, a fair SSA economic system would be the one that distributes goods to SSA individuals in proportion to their efforts (30). Effort typically comes in the form of productivity, ability, or talent. More effort in an economic activity ought to be rewarded more, and vice versa. A range of economists have offered several concepts to explain the notion of equity in health care and these have been summarized (8). These explanations emerged from the work done in the United Kingdom's National Health Service, and they all lead to the concept of “equal access for equal need” as a guiding principle for resource allocation. Olsen argued that this approach is egalitarian because it emphasizes an equitable distribution of health profiles (31).

## Normative Frameworks From Economics For PS

In economics, the explicit criteria that inform decision making may include opportunity costs, efficiency, and equity. The normative economic frameworks make use of explicit economic criteria to ensure efficient and socially desirable allocation of resources. Other criteria may include: equality; access; needs; and due process. Socially desirable outcomes are often viewed as equitable distribution of outcomes. Equality is often treated as the central principle for social justice. Rawls stated that it is “a standard whereby the distributive aspects of the basic structure of society are to be assessed” (22). The challenge, however, is how to specify it the normative frameworks: N-C Welfarist framework or the Non-Welfarist ones.

### N-C Welfarist Normative Economic Frameworks

The N-C welfarist framework is deeply rooted in consequentialism—a moral theory which holds that, a morally right action or rules, and such actions are to be evaluated in terms of their utility—good outcomes or consequences, only (4). Under different normative frameworks, such as the N-C welfarist approach or the non-welfarist approach, arguments in the SWF can be defined in different ways. Furthermore, the aggregation of these welfares can take different forms. Hurley summarizes the key features of the N-C Welfarist approach to normative economics to include the following (32): Welfarism; consumer sovereignty; utility maximization (behavioral assumption); and consequentialism. Welfarism relies on the notion that social welfare is only a function of individual utilities which are derived from only goods and services (32, 33). Utility is defined by revealed preferences which are assumed not to be distorted by ignorance, imperfect foresight, or misinformation because the individuals are “rational” and “responsible” in their choice making (17, 34).

The N-C Welfarist framework further assumes that individuals are the best judges of their welfare (consumer sovereignty) because the market is perfectly competitive. It advocates the reward of individual effort through the market system and it ignores the issue of the overall distribution of resources. This assumption ensures that the free market mechanism is accorded the supremacy to allocate resources efficiently. The policy implication is that with full information and no consumption externalities, consumers are able to maximize their utility (8, 35). However, the N-C welfarist framework for allocating resources is rejected by the non-welfarist approaches on ethical grounds.

### Non-welfarist Normative Economic Frameworks

The non-Welfarist frameworks are referred to as extensions beyond Welfarism, and include those approaches that allow non-utility attributes to be used to define the SWF. These approaches may include extra-Welfarist, communitarianism, capability approaches, decision-making approaches (DMA), and others. The development of a non-welfarist framework arose as it became clear that the “merit goods” argument, initially raised by Musgrave in his Theory of Public Finance, could not fit in the N-C welfare framework, necessitating departure from N-C welfarism (36). Attempts were then made by some economists such as Culyer (37) to introduce non-utility attributes into the Welfarist framework but were generally unsuccessful. This led to the development of non-Welfarist frameworks—referred to as extensions beyond Welfarism. These include all those approaches, excluding the N-C Welfarist approaches, which allow non-utility attributes to define the SWF. These approaches may include capability approaches, Extra-Welfarists, communitarianism, decision-making approaches (DMA), and Social Justice frameworks. These approaches themselves markedly differ from one another, but all share one thing in common: that they are not Welfarist (38).

The influence of the capabilities approach on the health care sector started following Sen's notions of functioning and capabilities theory (39). The result was the development of the extra-welfarist approach within health economics. Extra-welfarism is defined as a normative framework which supplements traditional welfare in the SWF with other “non-goods characteristics” of individuals (40, 41). In health care, the relevant characteristic is health and implies that the health status information directly influences the social states that individuals prefer, contrasting sharply with welfarism (18). The function may be defined as:

$$\begin{array}{l} \text{SWF}=\text{f}\left({\text{u}}^{1},{\text{u}}^{2}\ldots {\text{u}}^{\text{i}},{\text{v}}^{1},{\text{v}}^{2}\ldots {\text{v}}^{\text{i}}\right) \end{array}$$

Where u^i^, and v^i^ are the utility and non-utility attributes of the ith individual's consumption of goods and services respectively.

Sen had explained that “functionings” might include basic human functions such as “moving, being well-nourished, being in good health, being socially respected” (42). He then described “capability” as the extent to which a person is able to function in a particular way, whether or not he or she chooses to do so (43, 44). For example, according to Culyer, two pivotal concepts that emerge from the characteristics of people (capabilities approach) are “deprivation” and “need” (40). He stated:

- “If the characteristics of people are a way of describing deprivation, desired states, or significant changes in people's characteristics, then commodities and their characteristics are what are often needed to remove their deprivation.”
- Characteristics of people refers to attributes that describe a person and these may include their genetic endowment of health; his or her SES; moral-worth and deservingness; utility; severity of pain; and/or equity, fairness and social justice.

It is the non-utility characteristics, such as deprivation and need that create the demand for healthcare. The focus on deprivation and need implies that need and equity determine the demand for a health intervention, and not necessarily the willingness to pay as advocated in the N-C welfarist approach. Culyer advocated the use of Quality Adjusted Life Years (QALYs) as the unit of measure of health, and the weights used in the QALYs are not necessarily derived from utility values (40). The weights could be based on any factor (such as age, social/occupational roles/family responsibilities, initial health status, etc.) that affects how individuals value health for a person with the characteristic as opposed to a person without. Other authors have argued that other important considerations other than QALYs should be included as attributes of the SWF. The policy implication is that health is the key outcome in the SWF as opposed to utility, and equity attributes based on the key characteristics of people should be included in these outcomes as non-utility attributes of the SWF.

Mooney and Russell argue that communitarianism advocates for the use of community preferences in the SWF, and weights to attach to these preferences should be determined by members of the community (25, 45). Further, the approach emphasizes the need to differentiate individual from community preferences and proposes that the preferences of the disadvantaged groups should be used to address their disadvantage. In the communitarian approach, the theory aims to attain some degree of coherence and transparency in the assessment of equity from an economics perspective. It classifies reasons for a person's claim for a good into two sectors: one being “claims,” and the second being “other reasons” (46). “Claims” are defined as reasons backed by a notion of duty for one's claim for a good. Equity is seen to be consistent with community perspectives on how individuals should be treated relative to one another. Communitarianism is especially appealing to SSA health. This is because the sense of the community and community competencies are properties of the community, and it is therefore questionable whether aggregating the effects on individuals can capture the full benefits of community action. Thus, equity is defined by how claims are established, and then how different claims are weighted.

### Social Justice Frameworks

Social justice is defined in terms of the distribution of wealth, opportunities, and privileges within a society. Egalitarianism focuses on the equality of outcomes and states that the fairest allocation of resources in the health system is when benefits and costs from the allocation are distributed equally among all people as a key objective to achieve (47). This theory, however, ignores differences in effort, talent, and productivity when resources are being allocated. When all of society receives the same opportunities, then the rights of various groups within it, for example equality of men and women, can be realized (48). This is because equal outcomes are virtually impossible to achieve in practice due to differences in natural endowments, differences in people's capacities to benefit, and differences in people's willingness to participate because their tastes and preferences are not homogenous (30, 49). In practice, however, equality of opportunity should be pursued along with other social justice objectives. Thus, social justice policies should be judged by the equality of opportunities and/or equality of access accorded to the members of society. These policies should be pursued in conjunction with other social justice objectives such as human rights and the basic health needs (48).

The needs principle gives priority to members of society with the basic minimum of services which they need or are essential for the tolerable living (30, 50). In SSA health, this implies that more resources are needed to raise the health status of community members to a level comparable with people from non-SSA. This would be consistent with their governments' pledges to achieve SDG3. However, the needs principle is sometimes criticized because it does not recognize differences in productive contributions, or distinguish between real needs and manifested needs. For example, Rawls' difference principle has been criticized by many authors such as Arrow (51) and Harsanyi (52) for failing to recognize that people may also seek to maximize other outcomes and not just the distribution of goods and services (24, 30). Additionally, the needs principle does not provide an explanation for resource allocation beyond achieving the minimum standards required for human existence. Indeed the evidence in the literature suggests that although people care about need and show concern for the least disadvantaged, they also care about adverse effects from basing allocations solely on need, thus rejecting need as the sole foundation for a system of distribution (30).

### Specifying the Social Welfare Function in Practice

To specify the arguments for the SWF, two approaches are commonly used to elicit arguments that enter the SWF: (a) using a democratic process (using economic criteria, market criteria, or voting in a referendum) to elicit individual preferences and then aggregating them; or (b) imposing it on society—dictatorship (using government, experts, or community/opinion leaders).

Under the democratic process, three major approaches to specifying the SWF may be used: subjective approach; basic axiomatic approach; and moral justice approach (9, 17). The subjective approach completes the functional form of the SWF on subjective ethical grounds. Mishan explains that Bentham and colleagues argued that, social welfare ought to be the sum of individual utilities with a weighting of 1—utilitarian functions (17):

$$\begin{array}{l} \text{W}={\text{U}}^{1}+{\text{U}}^{2}+\ldots +{\text{U}}^{\text{n}} \end{array}$$

Where U^n^ is utility for individual n, and the policy qualifies for implementation when W > 0.

Sugden and Williams advocated for the use of directly obtained or inferred values of decision-makers in the specification of the SWF (53). Two methods exist for determining the decision-makers' objectives: government policy documents/guidelines; and carefully elicited/inferred objectives from surveys. There are, however, disagreements amongst the proponents of the subjective approach on the specification of the SWF from the democratic process (9). The disagreements stem from the fact that others have advocated for “normal” distributional judgments and yet what is “normal” is not defined. Some they have advocated for inequality aversion and yet there was not an agreement on the appropriate level of inequality aversion (9). So, other economists pushed forward the use of axiomatic approach.

The basic axiomatic approach, uses mathematics to investigate the existence and form of SWF (9). In response, other authors have suggested that it was the intensity and not the simple rankings that mattered when individual preferences were being aggregated into a SWF. Still others have suggested that the axiom on “rankings are independent of irrelevant alternatives” ought to be dropped and certain kinds of rank-order voting should be employed (54, 55). This would provide the analyst with the ability to weigh the gains of the winners against the losses of the losers. Just et al. argued that the major practical problem with the axiomatic approach was that, even under weaker conditions where voting works, transactions costs of voting and compiling the ranking would be prohibitive (9).

The proponents of Axiomatic approach argued that, the SWF is based on a set of plausible underlying axioms about individual preferences. These include:

- The domain of decisions is unrestricted;
- The Pareto principle applies; and
- Rankings are independent of irrelevant alternatives.

The Arrow Impossibility Theorem, however, proved that it is difficult to find a rule that satisfies all of the above properties when aggregating preferences into a SWF, unless the decision-making process is a dictatorship. Arrow's Impossibility Theorem states that if a social decision mechanism satisfies all the three assumptions of the SWF, then it must be a dictatorship: all social rankings are the rankings of one individual (54, 55).

The moral justice approach argues that the Arrow impossibility theorem happens as a result of the majority groups acting selfishly—preferring to eliminate consideration for the minority groups. To address this problem requires admitting moral considerations such as impartiality and economic justice in the SWF. Impartiality stands for the Moral concerns for equal treatment of individuals. In these SWFs, a criterion of distributional optimality is suggested which tends to advocate for equality or equal weighting. Slesnick explained that it was Amartya Sen who demonstrated that relaxing the interpersonal comparability assumption expands the spectrum of possible SWFs dramatically. Champions of the moral justice SWFs include Rawls, Harsanyi, Benthamite, Arrow, and others (9, 17).

Efforts to develop a generally accepted SWF have not been successful because there is no objective way of making interpersonal comparisons of individual utilities. In practice, a SWF requires the individual utilities to be cardinally measurable so that intensities of preferences can be compared. In contrast, the Pareto and compensation criterions, in which utility is measured in ordinal units, has many applications but is not useful for identifying a unique social optimum.

## Implications For P/S in Achieving SDG3

In making choices for investments in healthcare, it is imperative to have an efficiency criterion as a key principle that guides allocation of resources. Since the markets in health care do not clear and fail to address fair distribution of resources, government has to intervene. However, for government allocations to achieve fair and efficient allocations, there is need for evidence to be used in its priority setting process. Selecting interventions and policy instruments which are efficient and achieve social justice, makes priority setting desirable and fair exercise to achieve SDG3. To do so requires a normative framework that incorporates economic principles and social justice principles.

### The Preferred Normative Framework

The preferred framework for resource allocation to achieve SDG3 would be one that would have the potential to address economic issues (efficiency) and social justice issues (equity), resulting into a fair and just outcome of health maximization (24). Such a framework would allow social justice principles to be included either as policy objectives or as variable in the SWF. In practice, the policy objectives could be based on equality and maximization, according to individual/community characteristics. The principle that ought to guide procedural justice in SSA health should be viewed as: the level of people's involvement in the priority setting process; or the extent to which SSA citizens exercise autonomy in determining how resources are spent, which is an objective in its own right (30, 56). It is also consistent with the notion of self-determination or community control. This concept of autonomy in the input to resource allocation is also justified in the sense that it ensures that the community is appropriately informed of the potential health consequences of its choices.

The debate about the notion of social justice mirrors the wider debate within moral philosophy over what the appropriate criterion to achieve fairness in allocation of resources (25, 26). Should it be equity (27) or needs principles? In practice, we need a priority setting framework that incorporates both economic and social justice principles. Such a framework generates efficient outcomes/interventions and takes into account social justice principles. The choice of one form of justice, such as equality of access, can mean sacrificing another form of justice, such as unequal treatment of unequals. At the same time, equality of outcomes and very closely related to equality of opportunities or access can form government objectives that can be targeted by investments to turn into outcomes. For example, the presumption of equality provides an elegant procedure for constructing a theory of distributive justice using the following policy questions (47).

When need is defined by the severity of the disease alone, rather than the existence of the disease, it tends to give priority to those who are severely affected by an illness over those less severely affected, a maximin principle under Rawlsian theory. In this case, those clinically worse off, would get priority treatment, irrespective of the forgone improvements to the less ill. Thus, people with the worst initial health level would be given priority in the allocation of resources in this model. The third form of need is defined by the existence of an effective intervention, often referred to as the “capacity to benefit” by those targeted by the intervention approach (19, 57). It measures the extent to which those people affected by a health intervention itself would benefit from that intervention, by focusing on health gain as opposed to pre- or post-treatment profiles of the populations. The non-welfarist normative economic framework advocates this approach because it involves an epidemiological assessment of a health problem, and the existence of an effective intervention targeting such a problem. It is therefore a preferred concept of benefit for use in health, since it has potential to allow the selection of interventions that are effective. However, the capacity to benefit (effectiveness) is not a well-described concept for many interventions. It is therefore necessary for increased research to be undertaken in the “measurement of the notion of need,” in particular, in the measurement of “need” in SSA health. Thus, such a normative framework would take the form below. SWF = f (U_1_, U_2_, – – – –U_i_, V_1_, V_2_, – – – – –V_i_, W_1_, W_2_, – – – –W_i_)

Where U_i_ stands for the ith utility, V_i_ stands for the ith non-utility attributes and W_i_ stands for any other ith social justices principles not captured by U and V.

In practice, this would involve aggregating the function of the framework to take two forms:

- SWF = f (health attribute) + f (other attributes) + f (equity) + etc. In this case, if the function of the health attribute is zero, the intervention still has a score, or
- The SWF = (health attribute) [f (other attributes) + f (equity) + etc.] In this case, if the health attribute is zero, then the intervention would score zero thus– not a healthcare interveniton.

Given the arguments by Richardson of laundering society's preferences and Mooney's Claims (communitarian arguments) from the community members affected by the intervention, it would only be fair that the form of the functional form of the intervention would be determined by the community preferences.

### Generating Economic Evidence to Support P/S

The common approach to assessment of social welfare improvement (economic evaluation) in Paretian economics is the use of the Kaldor-Hicks efficiency criterion to assess costs and benefits of various options. These benefits (utility or other measure) and costs are then combined to ascertain the overall benefit relative to the costs using Cost Benefit Analysis (CBA) (13, 38, 58). Economic evaluation of health care stems initially from standard Paretian Welfarist views and it follows the basic principles of consumer sovereignty and Pareto optimality (6, 13, 59). In CBA, for example, benefits are measured in monetary units and are based on individuals' willingness to pay for a service and it is theoretically relevant to the compensation principle as the means to achieve both absolute and relative efficiency.

The monetary unit of measure of outcomes such as in CBA overcomes all the weaknesses of the preceding units of outcome measures. However, for health outcomes or benefits valuation in health care—especially in life and death situations, two clear difficulties arise with the use of money metric for measuring welfare in CBA. An example of such valuations is the QALYs. Quality Adjusted Life Years express the sum of individual utility gains from medical interventions elicited using subjective valuation techniques such as time trade-offs. As such, they are potentially a useful measure in deciding how to allocate resources. They should not, however, be equated with the overall value that society places on different health care programmes. The QALYs that society places on different health care interventions is determined by aggregating the number of QALYs gained, but also by a series of distributional and ethical considerations (38). In most cases, the QALYs may be weighted to account of the distributional and other ethical issues before the aggregation takes place (39). This should be made clear in the presentation of QALY-calculations, particularly in cost-per-QALY league tables.

Thus, benefit from an allocation could be measured using one, or a combination, of the three broad units: the natural unit (e.g., life years), subjective values (quality of life), or monetary values (willingness to pay) of the services distributed in the community. Using economic evaluation to measure the relative efficiency of a policy or intervention, demonstrates that all these frameworks, have limitations because of the units employed for valuing benefits/outcomes. For example, when the natural units are used as measures, the results cannot be used across different disease interventions. The use of QALYs overcomes the problem of comparative analysis across different disease interventions and can therefore be used for comparative analysis across the whole health sector but cannot be used outside the health sector. Moreover, issues of measuring the quality of life remain unresolved.

### Generating Non-economic Evidence

SSA health policy objectives should be informed by their ordinary people's values and aspirations identified through empirical research. This would involve ethical values being subjectively derived from a sample of each countries' population and then being subjected to ethical analysis and criticism. These values can then be quantified and converted into a scale that can be used to weight the importance of different SDG3 outcomes or some other chosen measure of health improvement. In the event that the social value is shown to lead to abhorrent outcomes, such as racism, sexism, or discrimination, then preferences should be “laundered,” and an iterative process undertaken between the ethicist and the public.

For priority setting purposes, Richardson argues that social ethics ought to guide the government health policy objectives, and these ethical issues should be captured using the “empirical ethics” approach. These objectives should be subjected to ethical debate so as to reflect the views of the citizens (60, 61). Subjective values (pleasure, utility, or health) would be elicited using the empirical approaches, since subjective valuations are widely accepted and well-established (30). The cardinal measurement of the elicited values would be undertaken to specify the intensity of preferences, assumed to be the same finite end points, 0–1, on the cardinal health scale. The cardinal measurement would also allow for the elicited preferences to be interpersonally compared to avoid the Arrow impossibility theorem (31, 61). Two approaches are preferred for eliciting public views on health objectives: empirical ethics and communitarianism.

The first issue is related to the distribution of benefits—equity concerns. Knowing that measuring welfare using the willingness to pay concept is determined by the ability to pay, the allocation of health care resources would be “skewed” toward the wealthy. Empirically, van Doorslaer et al. found that among 10 Organization for Economic Co-operation and Development—a loose organization of 30 countries such as Australia, the United Kingdom, the United States, and others sharing a commitment to democratic government and the free market economy. Countries included in their analysis, there was strong support for equity in health care as indicated in their policy documents (24). Also people have been consistently found to be prepared to sacrifice total benefits to achieve equity, even when they knew that they would be in the group which would be hurt by the redistribution. There are several processes for empirically determining the population ethics of resource allocation including: “communitarian claims” by Mooney (46).

The second issue involves the discomfort society has in valuing life and death issues in monetary terms, resulting in its reluctance to participate in such exercises. This has led to the emphasis on the use of the natural units such as numbers and/or percentages of the outcomes of interest, and the focus on subjective valuations of individual welfare/benefit. Subjective valuations are undertaken using instruments such as rating scales, standard gamble, and time trade-offs to assess the relative severity of the consequences of the diseases. In these valuations, characteristics of severity of diseases such as pain and disability are determined through repeated assessments over time to establish the durations. The data derived may be expressed over an interval scale to estimate the intensity, which would then be attached to the attributes or criteria in Normative Framework.

### Limitations of this Study

However, achieving SDG3 requires addressing the whole of the economy since health is affected by issues from all the other sectors of the economy. This study focused on SDG 3, which means that the normative framework identified may be restrictive and only applicable to health sectors. Further, this study did not explore issues in the funding of healthcare, took the organization of healthcare as given and so it did not identify the most appropriate form of reform of the healthcare system to achieve the SDG3. This study ignored the structural and other issues in the delivery of health in SSA countries. It focused on normative frameworks and its principles thereof which ought to inform the decision making process in the health sector to achieve the SDG3.

## Summary

In SSA, there are lots of health challenges which requires significant investments. Government ought to have a dominant role in the mobilization of funding resources and regulation of healthcare services, since it has more ability to compare the claims of every one in society, ceteris paribus. The decision context is such that priority should be accorded to those in higher “need” and should reflect social values. Inclusion and recognition of community members as legitimate claimants of the resources allocated would allow communities to determine what weights should be attached to the stated arguments in the SWF. Therefore, the normative framework ought to allow the concept of benefit to reflect the stated policy objectives being pursued.

In SSA where health problems outstrip available resources, it is important for any priority-setting framework designed to achieve SDG3 by maximizing outcomes given the limited budgets. Using economic evaluation evidence assists decision-makers to identify such options for change which, offer value-for-money from investments. The appropriate normative framework should include efficiency and social justice principles. Adoption of Non-Welfarist normative economic framework which, allows utility, health, and other attributes important to the community to enter the SWF is what ought to be done. The SWF should be specified empirically by the decision-makers and the community representatives. Such a function ought to recognize the opportunity costs as a principle, marginal analysis, efficiency, and equity, regardless of how they are defined in order for the decisions makers to choose those strategies, which maximize health outcomes for a given resource allocation.

In conclusion, both economic evidence and social justice principles (distributive and procedural), ought to inform priority setting so as to improve SDG3. The social justice principles which ought to be included in the framework may include equity (both vertical and/or horizontal equity); need (in whatever form it is defined); fairness (in whatever form it ought to be defined, either as distribution of outcomes, autonomy or community control); and/or access (both physical and perceived forms). Such principles may enter the SWF directly, or may stand independently as social/policy objectives which can be pursued to achieve the SDG3. The priority-setting framework that SSA ought to adopt to improve the SDG3 therefore ought to be non-economic normative framework with both economic and social justice principles, regardless of the level of rigor.

## Author Contributions

MO led the conception and design of the study, reviewed the files where the data for analysis and interpretation was generated from, and drafted the manuscript. AA, JM, and WG read the draft, reviewed the findings, and worked on the manuscript. All authors critically analyzed the paper for important intellectual content, read and approved the final manuscript, contributed to the preparation, and writing of the paper.

## Conflict of Interest

The authors declare that the research was conducted in the absence of any commercial or financial relationships that could be construed as a potential conflict of interest.

## Abbreviations

SDGs: Sustainable Development Goals
SSA: Sub-Saharan Africa
P/S: Priority Setting
UN: United Nations
WHO: World Health Organization.
